# Graphene-Based Optoelectronic Mixer Device for Time-of-Flight Distance Measurements for Enhanced 3D Imaging Applications

**DOI:** 10.1021/acs.nanolett.3c00909

**Published:** 2023-06-16

**Authors:** Paul Kienitz, Andreas Bablich, Rainer Bornemann, Maurice Müller, Felix Thiel, Peter Haring Bolívar

**Affiliations:** Department of Electrical Engineering and Computer Science, University of Siegen, Hölderlinstrasse 3, 57076 Siegen, Germany

**Keywords:** graphene, time-of-flight (ToF), range sensor, distance measurement, superheterodyning, 3d imaging, optoelectronics, optoelectronic mixer, 3d sensor

## Abstract

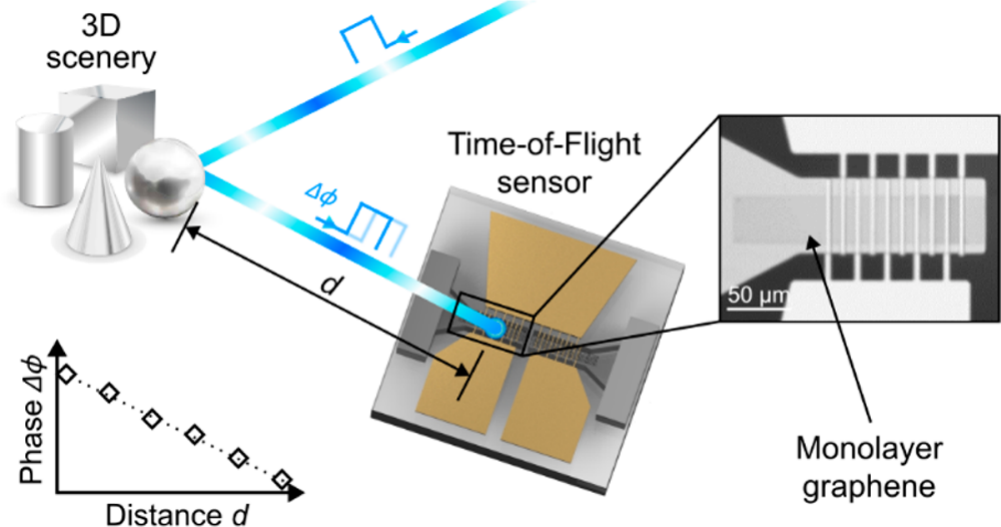

A large and growing number of applications benefit from innovative and powerful 3D image sensors. Graphene photodetectors can achieve 3D sensing functionalities by intrinsic optoelectronic frequency mixing due to the nonlinear output characteristics of the sensor. In first proof of principle distance measurement demonstrations, we achieve modulation frequencies of 3.1 MHz, signal-to-noise ratios of ∼40 dB, distance detection up to at least 1 m, and a mean accuracy of 25.6 mm. The scalable More than Moore detector approach enables geometrical fill factors close to 100% and can easily complement powerful functionalities by simple back-end integration on top of CMOS electronics.

3D vision is a fundamental cornerstone for highly complex tasks in the future fields of autonomous driving for scene recognition^1,2^ or novel manufacturing and quality tests for Industry 4.0.^3−5^ The medical sector,^6^ human recognition^7^ and scanning,^8^ and scene analysis in security applications for crowd screening^9^ can benefit from high-performance 3D camera systems as well as the entertainment sector,^10,11^ which pioneered widespread 3D imaging systems and awareness with the first generation of Microsoft’s Kinect.^12^ Because most applications differ in their requirements, research and industry developed various depth measurement and 3D imaging concepts, each with its own advantages and challenges. However, the objective of all systems is to improve overall performance characteristics, especially lateral precision and resolution, speed of image acquisition for real-time applications, reliability, and integrability, e.g., for mobile or space-limited systems.

State-of-the-art 3D imaging systems typically use either triangulation^13,14^ or the time-of-flight (ToF)^15^ principle. Systems based on triangulation utilize geometries and special configurations to compute depth information from optical data. These systems are subdivided into (I) passive systems, such as stereo vision^8^ or dynamic vision^13^ entailing significant drawbacks and limitations in demanding applications, and (II) active systems that compensate for many of these limitations. The structured light approach, for example, uses specific light patterns to illuminate the scene, using diffraction of the reflected pattern to compute depth information, achieving depth resolutions of at least a several tenths of a micometer.^16^ However, triangulation-based systems inevitably require multiple active elements, preventing highly integrated devices or miniaturized systems. This approach is also error-prone, as it has difficulty detecting uniformly shaped or monochromatic objects.

ToF methods such as the light detection and ranging (LiDAR)^17^ or photonic mixer devices (PMD)^18^ exploit the finite speed of light and utilize the difference between light emission and detection of photons reflected from the scene. The propagation time results in the distance. ToF principles range from (I) direct methods to accurately measure propagation time requiring very precise timing and fast sensors, (II) indirect methods that modulate the emitted light and measure the phase delay due to the propagation time, and (III) frequency modulated methods^2^ that allow depth resolutions in the range of a few millimeters for less complex devices^19^ down to several tenths of a micrometer for very complex and time-consuming techniques.^20^ These approaches allow for very large-scale integration, e.g., enabling 3D in mobile applications, and do not suffer from scene-dependent uncertainties such as objects. However, today’s approaches rely on highly complex sensor architectures or systems, such as scanning systems involving sensitive micromirrors, linked with extensive readout complexity and signal processing (e.g., mixing and circuitry).

The claim of this paper is the use of graphene^21^ for indirect ToF paving the way to overcome bandwidth limitations of silicon-based technology and simplifying the sensor architecture by exploiting nonlinearities resulting in internal optoelectronic frequency mixing. Graphene can exhibit charge carrier mobilities above 200 000 cm^2^ V^–1^ s^–1^,^22^ allowing bandwidths far beyond that of state-of-the-art silicon devices. Furthermore, the linear dispersion relation of graphene allows for an unlimited choice of illumination wavelengths and thus does not restrict applications due to light or laser safety limitations.^23^

Although mixing of two optical^24^ or two electrical signals^25^ has already been demonstrated in graphene as well as optoelectronic mixing at modulation frequencies above 65 GHz^26,27^ in highly optimized device structures, previous articles only mention 3D imaging to be a potential application lacking further demonstration.

In this work, we demonstrate for the first time a metal–graphene–metal photonic mixer device (MGM-PMD) for optical distance measurements exploiting internal optoelectronic frequency mixing. Proof of concept measurements show that operation frequencies in the megahertz range can easily be achieved without relying on complex device architectures or e-beam lithography.

Conventional PMDs require a highly precise gate control and complex readout circuitry (I) to separate charge carriers between two integrated readout diodes and (II) to detect a phase shift of the incoming amplitude-modulated light. An electrical circuit-based cross-correlation of both the modulated gate and the optical signal reflected from the scene results in 3D sensing capabilities. In contrast, the PMD functionality demonstrated in this work is provided by the MGM-PMD itself because an intrinsic frequency mixing of the electrical gate modulation with the optical modulation^27^ occurs due to nonlinearities in graphene. Besides reduced integration efforts (no differential readout of two diodes is necessary), the complexity of an electrical circuitry for signal generation and to detect and evaluate accumulated charge on the respective readout diodes is significantly reduced. In the following section, the device technology and fabrication steps are presented.

Figure 1 shows the process chain of the MGM-PMD fabrication. At first, trenches for the buried gate contacts have been wet chemically etched into the glass substrate (Figure 1a) followed by chromium (Cr) gate contact deposition (Figure 1b) and aluminum oxide (Al_2_O_3_) passivation (Figure 1c). In a next step, interdigitated metal finger structures consisting of chromium/gold (Cr/Au) have been deposited (Figure 1d) prior to the monolayer graphene transfer (Figure 1e). Finally, a further Al_2_O_3_ passivation layer has been deposited on top of the MGM-PMD (Figure 1f) in order to protect the device from environmental influences, which, for example, cause unwanted doping.^28^ Further details on the MGM-PMD fabrication are given in the Methods section. A false-color Raman mapping image of the metal–graphene–metal photodetector on a glass substrate (purple) with graphene channel (cyan), source and drain Cr/Au contacts (yellow), and the buried gate Cr contact (black) without passivation layer is shown in the close-up (black rectangle) in Figure 1.

**Figure 1:**
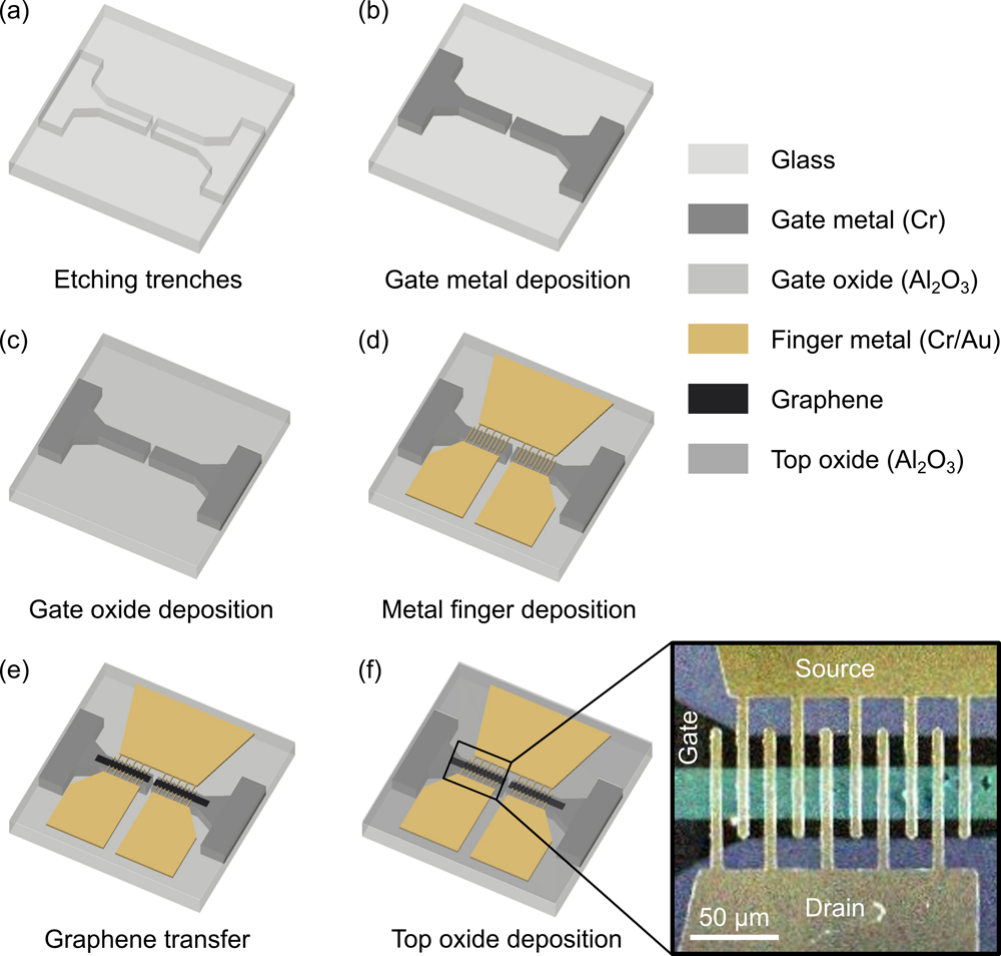
(a–f) Schematic representation of the technological fabrication steps for the MGM-PMD. The close-up (black rectangle) shows the Raman mapping image in false colors of a metal–graphene–metal photodetector on a glass substrate (purple) with graphene channel (cyan), source and drain Cr/Au contacts (yellow), and the buried gate Cr contact (black). Raman analyses were performed without Al_2_O_3_ passivation to obtain a low noise image.

2D material qualities (crystallinity and defect density) have been monitored by Raman spectroscopic analysis (cf. Figure 2a). The small D-peak indicates a low defect density in monolayer graphene stating the good initial graphene quality and thorough PMMA-assisted graphene transfer.

**Figure 2:**
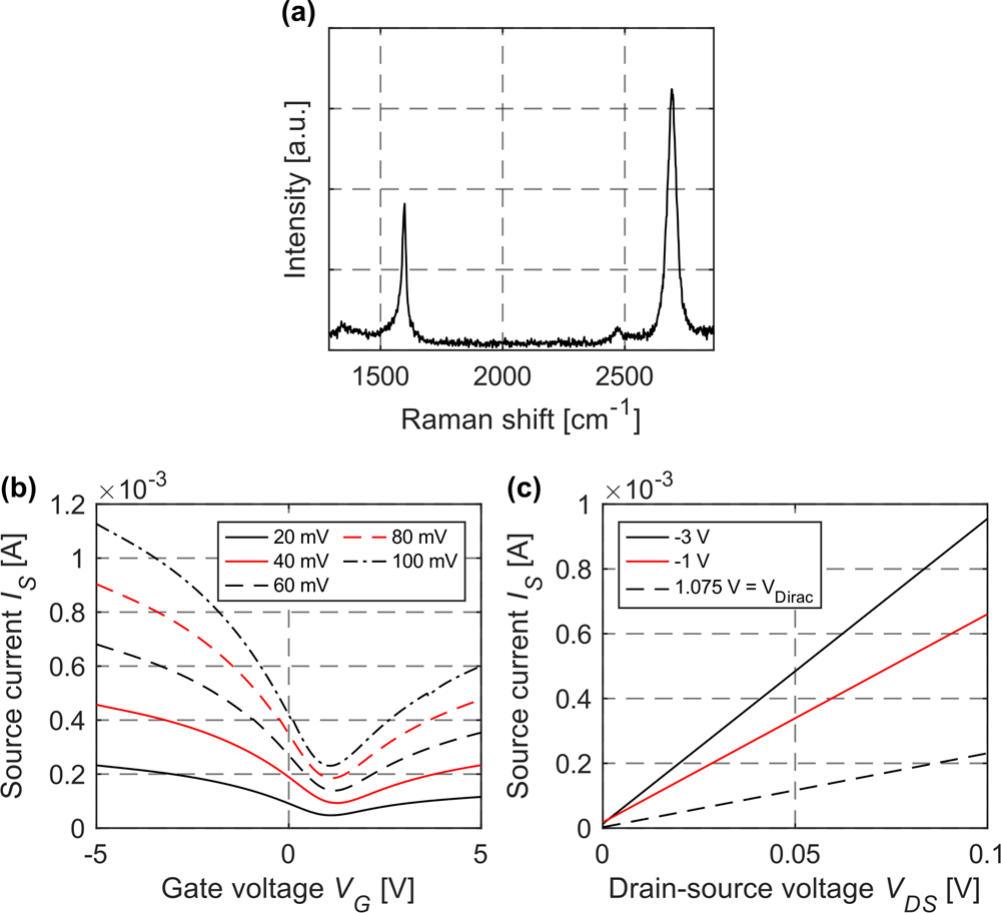
(a) Raman spectrum of the graphene channel. The peaks characteristic for p-doped graphene^29,30^ (G-peak at 1598 cm^–1^ and 2D-peak at 2693 cm^–1^) are clearly visible. The low intensity of the D-peak at 1348 cm^–1^ indicates a high quality of the graphene with few defects. The excitation wavelength of the laser is 532 nm. (b) *I*_S_–*V*_G_ curves for drain–source biases of *V*_DS_ = 20 to 100 mV (Δ*V*_DS_ = 20 mV) and (c) *I*_S_–*V*_DS_ curves for gate biases of *V*_G_ = −3 to 1.075 V.

Prior to the distance measurements, current–voltage (*I*–*V*) measurements were performed to electrically characterize the devices. First, the source current (*I*_S_) has been determined to be a function of the gate voltage (*V*_G_) (Figure 2b) showing the expected nonlinear behavior. From the results presented in Figure 2b, charge carrier mobilities have been extracted with the direct transconductance method (DTM) as proposed by Zhong et al.^31^ considering the GFET as a parallel connection of transistors. Taking this consideration into account, the effective channel width *W*_eff_ is given by the channel width *W*_Ch_ multiplied by the number of gaps between the fingers (details on the finger structure are given below). Utilizing the DTM, electron and hole mobilities of μ_e_ = 140 cm^2^ V^–1^ s^–1^ and μ_h_ = 266 cm^2^ V^–1^ s^–1^ have been extracted for *V*_DS_ = 100 mV, respectively. Compared to conventional semiconductor or other graphene-based devices, the mobilities presented in this work are comparatively low because CVD graphene has been used in conjunction with additional encapsulation of graphene in between Al_2_O_3_ and SiO_2_ that reduce mobilities further. Furthermore, source current vs drain–source voltage (*V*_DS_) curves have been acquired, showing resistance behavior (Figure 2c). For the first-ever distance measurement demonstration, the gate has been modulated because the behavior of *I*_S_ as a function of *V*_G_ is highly nonlinear (Figure 2b), whereas *I*_S_ linearly depends on *V*_DS_ (Figure 2c). Previously, it has been shown that *V*_DS_ modulation can also be used to generate optoelectronic mixing in a graphene device, but at considerably higher channel bias voltages of at least 3 V and optical power exceeding 20 mW.^32^

In order to estimate device and design specific modulation frequencies prior to experimental 3D measurements, the gate–drain, gate–source, and drain–source capacitance of the structure have been determined in a material- and architecture-specific manner. In this context, the drain–source capacity *C*_DS_ for MSM finger structures can be estimated within 2% accuracy for the sensor geometry presented here taking into account the following relationship:^33,34^
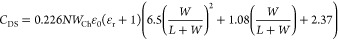
1where *N* is the finger pair number, *W*_Ch_ is the width of the graphene channel, ε_r_ is the permittivity number, *W*_F_ is the finger width, and *L* is the finger spacing. This interpolation considers the layer thickness of the contact material *t*_Con_ to be much smaller than the finger width *W* (*t*_Con_ ≪ *W*). The drain–source capacitance of the MGM-PMD prototype is *C*_DS_ = 7.2 fF (*N* = 5, *W*_Ch_ = 29.02 μm, ε_r,Graphene_ = 6.9,^35^*W* = 4.17 μm, *L* = 11.22 μm, *t* = 135 nm). According to Mao et al.,^36^ the gate–source/gate–drain capacitance is to a first approximation *C*_GS/GD_ = ∼12 pF (ε_r,Al_2_O_3__ = 9.5, *t*_Al_2_O_3__ = 20 nm). In this first ever demonstration, the modulation frequency of the graphene PMD achievable in the current design is significantly dominated by the gate capacitance. Considering the experimentally determined maximum sensor resistance of *R* = 427 Ω at the Dirac point, the 3 dB cutoff frequency is found to be *f*_g_ ≈ 31 MHz. According to this estimation, electrical and optical modulation frequencies have been chosen far below the 3 dB cutoff frequency in first distance measurement demonstrations utilizing graphene-based mixing devices to ensure sufficiently high signal-to-noise ratios of the sensor output signal as demonstrated in the next section (cf. Figure 4).

A schematic of the optical distance measurement setup utilizing 2D material-based MGM photodetectors is presented in Figure 3. The setup includes a 405 nm amplitude modulated laser, a motorized linear stage with a retroreflector, an electromechanical shutter, a function generator, a current–voltage amplifier, a lock-in amplifier, and a voltage source. The device under test (DUT) is contacted electrically using probe needles. The drain contact is biased utilizing a DC voltage supply to ensure charge transport across the channel region by spanning up an electrical field. The optical modulation signal *f*_opt_ from the function generator serves as an input for the laser source. The same signal is fed into a lock-in amplifier, which internally generates a difference frequency of *f*_opt_ and a freely adjustable frequency *f*_el_ that is provided by the lock-in amplifier itself. The signal *f*_el_ serves as the electrical modulation signal for the gate of the MGM-PMD. This measurement technique ensures that the lock-in amplifier reliably detects the differential frequency |*f*_opt_ – *f*_el_| being generated by the DUT. In a next stage, the optoelectronically mixed current signal of the DUT is amplified (gain 10^4^) and converted to a voltage which is then used as input signal for the lock-in amplifier. The motorized linear stage is used to move the retroreflector and utilized to change the distance from the sample to the light source. The laser beam can be blocked by means of an electromechanical shutter. A high-speed photoreceiver with a crystalline Si-PIN photodiode (iC212, iC-Haus GmbH) connected to a conventional oscilloscope is used to monitor the optically modulated signal.

**Figure 3:**
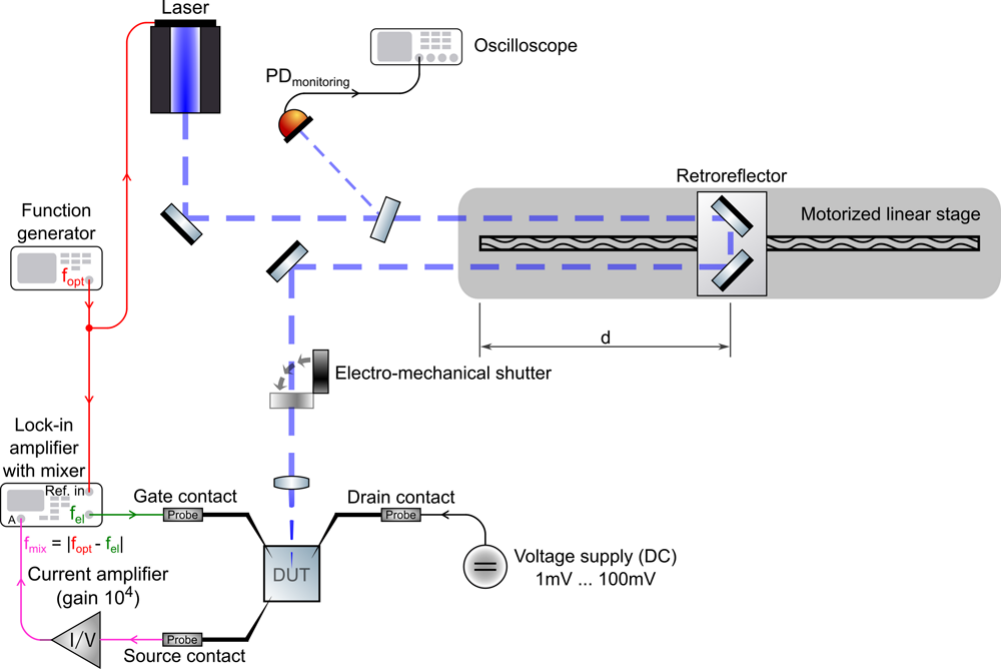
Schematic representation of the measurement setup for ToF distance measurements using a graphene-based MGM-PMD. In this configuration, the lock-in amplifier has been set to lock onto the difference frequency internally generated from the electrical modulation signal (*f*_el_) and the optical modulation frequency (*f*_opt_).

Figure 4 shows the time-resolved signal amplitude (top) as well as the corresponding phase ϕ (bottom) of the mixed signal (*f*_mix_ = 6 Hz) for an optical modulation of *f*_opt_ = 3 130 866 Hz and an electrical modulation of *f*_el_ = 3 130 860 Hz. In this experiment, modulation frequencies have been chosen to generate a mixed frequency component below 50 Hz to block parasitic environmental noise, especially mains hum. Both graphs depict a stable mixing signal with the shutter open for a fixed distance *d* = 0 mm (cf. Figure 3). The distance measurement has been conducted using a gate modulation voltage of *V*_G_ = 3 V_pp_ (DC offset *V*_DC_ = −2 V) and a drain–source bias of *V*_DS_ = 10 mV. From the incident laser power (*P*_laser_ = 10 mW) and a detected mixing current of 0.09 μA, a sensitivity of ∼0.1 mA W^–1^ for the mixed signal can be derived at that operating point. The signal located at the differential frequency *f*_mix_ exhibits an excellent phase stability of 0.56° corresponding to a depth resolution of ∼75 mm at 3.13 MHz with the shutter open. In time-of-flight optical distance measurements, a high phase stability of the sensor is a prerequisite to achieve a high depth resolution. The signal-to-noise ratio of the differential frequency components amplitude signal of the MGM-PMD exceeds values of 40 dB, indicating that in further experiments light intensities might be reduced or distances increased. In a next step, the nonlinear output characteristic of the graphene sensor has been exploited to demonstrate time-of-flight optical distance measurements.

**Figure 4:**
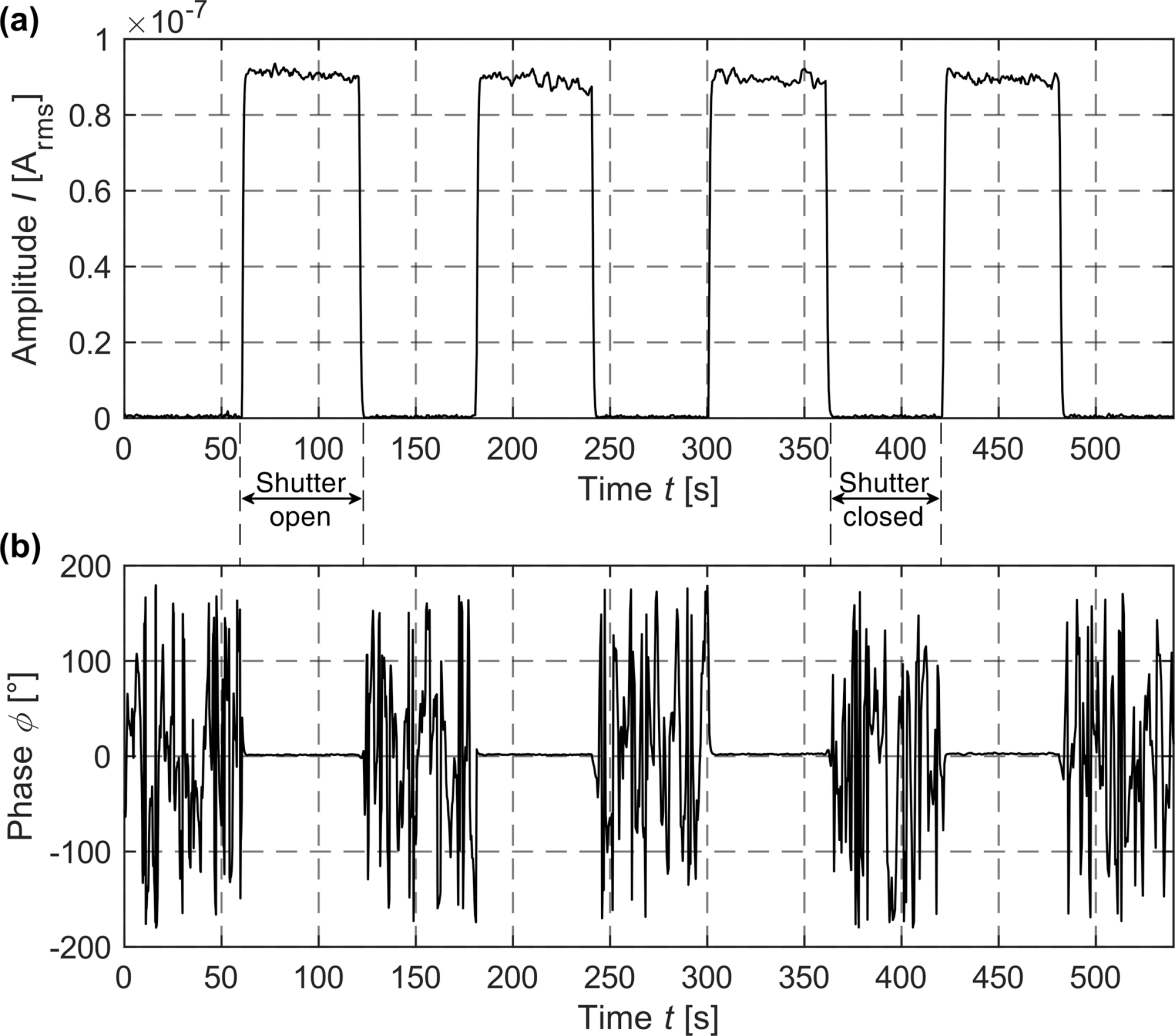
(a) Transient behavior of the mixed signal amplitude at *f*_mix_ = 6 Hz for an electrical modulation of *f*_el_ = 3 130 860 Hz and an optical modulation of *f*_opt_ = 3130866 Hz; (b) the corresponding phase with the shutter open (= stable phase) and closed (gate voltage *V*_G_ = 3 V_pp_ with an DC offset *V*_DC_ = −2 V, drain–source bias of *V*_DS_ = 10 mV, I–U gain 10^4^, laser wavelength λ_laser_ = 405 nm and irradiance *E*_e_ = 328 mW/mm^2^ (respectively, laser power *P*_laser_ = 10 mW)).

The distance measurement functionality of the MGM-PMD is verified experimentally in Figure 5, where Figure 5a represents the raw data and Figure 5b the mean values extracted from it. Here, the theoretical (expected) and the measured phase shift of the MGM sensor output are shown as a function of the distance *d*. As expected, the phase of the mixed signal is almost proportional to the distance, here shown for distances up to at least 1 m. The theoretical phase as a function of depth for ToF imagers has been calculated according to^37^
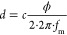
2where *c* is the speed of light, ϕ is the phase, and *f*_m_ is the modulation frequency. The mean accuracy,^2^ i.e., the deviation from the theoretical phase, is found to be 25.6 mm. The experimental results presented here clearly verify and demonstrate that the nonlinear behavior of 2D material graphene enables optical distance measurements by optoelectronic frequency mixing. The simple sensor design approach can achieve fill factors close to 100% whereas conventional time-of-flight detector concepts stagnate at 22%.^38,39^ A huge benefit is the simple back-end integration on top of semiconductor electronics or flexible substrates, enabling additional sensor functionalities and performance on-chip, making the MGM-PMD a More than Moore device.^40^ The detector presented here is thus a promising candidate to compete with alternative high-performance More than Moore devices in the future. The equipment used for distance measurement, such as function generator, lock-in amplifier, current amplifier, or stand-alone laser, can later be replaced by integrated devices. For example, if the differential frequency is fixed (defined by the two modulation frequencies *f*_el_ and *f*_opt_), the lock-in amplifier can be implemented by integrating either simple narrow bandwidth filters or more sophisticated phase sensitive detector (PSD) and low-pass filter below the sensor as part of the readout electronics.

**Figure 5:**
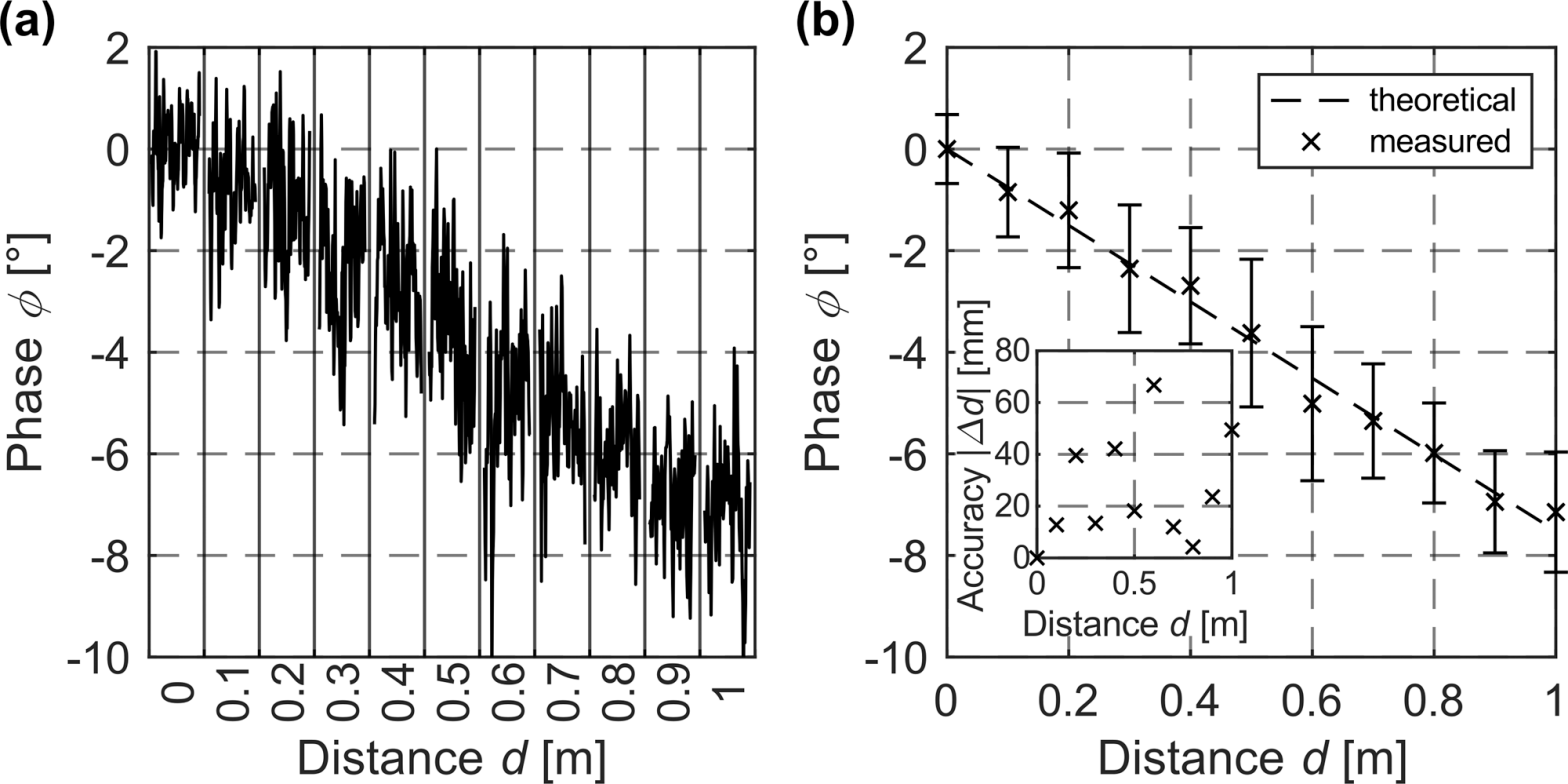
First experimental demonstration of a distance measurement using our MGM-PMD for distances up to 1 m. Distance-dependent phase shift of the mixed signal (*f*_mix_ = 6 Hz) for *f*_el_ = 3130860 Hz and *f*_opt_ = 3130866 Hz. The results were obtained at a gate voltage *V*_G_ = 3 V_pp_ (*V*_DC_ = −2 V), a drain–source bias of *V*_DS_ = 10 mV, a wavelength of 405 nm, an irradiance of *E*_e_ = 328 mW/mm^2^, and a laser power of *P*_laser_ = 10 mW. (a) Raw data of the phase for different distances. (b) Mean values including error bars for the raw data shown in (a). Inset: accuracy extracted from the experiment, with a mean accuracy of 25.6 mm.

In conclusion, intrinsic optoelectronic frequency mixing in graphene-based MGM-PMDs has been systematically investigated and utilized for optical distance measurements. The mixing effect thereby is enabled by the nonlinear output and transfer characteristics of the detector. The MGM-PMDs enable range measurements, hence 3D measurement capabilities, at modulation frequencies up to at least 3.1 MHz and mixing frequencies of 6 Hz in first proof of principle demonstrations. A distance of up to at least 1 m and an accuracy of ∼26 mm have been successfully demonstrated. A signal-to-noise ratio of ∼40 dB indicates that the current detection limit of 10 mW, corresponding to an irradiance of 328 mW/mm^2^, might be reduced in further experiments. Utilizing the innovative 2D material technology approach, sensor architectures and material compositions can further be developed towards fast, highly accurate, and sensitive distance measurement detectors and systems. The very simple device architecture and fabrication is scalable and allows for sensor integration on top of silicon electronics with fill factors close to 100%. This approach can enable significant performance improvements of 3D imaging systems compared to existing technologies.

## Methods

MGM-PMDs have been fabricated onto conventional glass substrates (D 263 T eco, SCHOTT). Prior to the contact deposition and graphene transfer, the substrates have been thoroughly cleaned. Subsequent patterning utilizes standard UV lithography (MJB3, SUSS MicroTec). In a first step, 100 nm deep trenches have been etched chemically by a buffered hydrofluoric acid (BOE 7-1 (AF 87.5-12.5) avec Surfactant) solution to define the buried gate contact areas. Next, 100 nm chromium has been deposited by RF sputtering at 13.56 MHz to fill the trenches followed by atomic layer deposition (ALD) to create a gate oxide layer of Al_2_O_3_ with a thickness of 20 nm. This gate oxide has been patterned and etched back in the gate contact area with BOE in order to contact the gates electrically. After that, source and drain areas have been defined in another lithography step. RF sputtering followed by a lift-off process has been applied to realize interdigitated Cr/Au (15 nm/120 nm) finger contacts. Single-layer graphene has been grown on top of a copper foil in a Moorfield NanoCVD-8G system by thermal assisted chemical vapor deposition. The graphene monolayers have been transferred by a conventional wet method using spin-coated poly(methyl methacrylate) (PMMA) as a support for the graphene. The 2D film has been separated from the growth substrate by etching the copper in sodium persulfate (Na_2_S_2_O_8_). The PMMA-supported graphene has been transferred onto deionized water for cleaning and fished out with the prepatterned target substrate. The PMMA has been thoroughly removed by a multistep process using ultraclean solvents. In a next step, graphene channels have been patterned and etched by reactive ion etching with an oxygen plasma. Finally, a passivation layer of SiO_2_/Al_2_O_3_ (5 nm/80 nm) has been deposited on top of the MGM-PMD graphene channel serving as a protective layer against ambient influences (the evaporated SiO_2_ is required as a barrier layer to avoid damaging the graphene during the ALD process).

Raman studies have been performed using a confocal microscope (TE 300, Nikon) and a laser (COMPASS 315M-100, Coherent) with a wavelength of 532 nm and a maximum excitation power of 100 mW. The signal has been collected through a 40× objective (NA 0.6, Nikon) and was detected using a monochromator (Triax 320, Horiba Jobin Yvon GmbH) and an EMCCD camera (Newton DU970P-BV, Andor Oxford Instruments).

Electrical device characterization comprises *I*–*V* measurements that have been performed using a SUSS microprobe station, connected to a Keithley 4200-SCS parameter analyzer.

Phase sensitive signals have been converted using a FEMTO DLPCA-200 *I*–*V* converter and amplifier and acquired via lock-in technique (SR865A, Stanford Research Systems). Distance measurements have been performed using an Omicrometer LDM405D.180.150 laser diode module with a peak wavelength of 405 nm. The power on the detector has been determined by a crystalline silicon reference detector (OP-2 VIS, Coherent) and a laser power meter (FieldMaxII-TO, Coherent).
